# Local gyrification index and sulcal depth as imaging markers of cognitive decline in Alzheimer’s disease

**DOI:** 10.3389/fnagi.2025.1635861

**Published:** 2025-08-12

**Authors:** Yongsik Sim, Changmin Seo, Young-gun Lee, Byoung Seok Ye, Ilwoo Lyu, Beomseok Sohn

**Affiliations:** ^1^Department of Radiology and Center for Imaging Science, Samsung Medical Center, Sungkyunkwan University School of Medicine, Seoul, Republic of Korea; ^2^Graduate School of Artificial Intelligence, Pohang University of Science and Technology, Pohang, Republic of Korea; ^3^Department of Neurology, Ilsan Paik Hospital, Inje University College of Medicine, Goyang, Republic of Korea; ^4^Department of Neurology, Yonsei University College of Medicine, Seoul, Republic of Korea; ^5^Department of Computer Science and Engineering, Pohang University of Science and Technology, Pohang, Republic of Korea

**Keywords:** Alzheimer’s disease, mild cognitive impairment, magnetic resonance imaging, cerebral cortex, cortical morphometry, gyrification

## Abstract

**Purpose:**

To investigate the correlation between cortical thickness (CT), sulcal depth (SD), local gyrification index (LGI), and cognitive scores in patients with Alzheimer’s disease (AD).

**Methods:**

A total of 200 patients with AD from 2014 to 2021 were included, confirmed by 18F-florbetaben-positron emission tomography, and having a Clinical Dementia Rating score of 0.5 or 1. Demographic and clinical data were collected, and cognitive function was assessed through the Mini-Mental State Examination (MMSE) and Seoul Neuropsychological Screening Battery (SNSB)-II, with specific *z*-scores used for multiple divisional cognitive functions. CT, SD, and LGI were extracted from the 3D T1-weighted images acquired with 3-T MRI scanners. General linear models were used to examine associations between cortical features and cognitive scores, controlling for age, sex, and years of education. Cluster-level significance was determined using a family-wise error (FWE)–corrected threshold of *p* < 0.05, with a cluster-forming height threshold of uncorrected *p* < 0.01.

**Results:**

The analysis included patients with a mean age of 73.7 years and an average MMSE score of 23.8. The cortical shape features of multiple brain regions showed significant correlations with the MMSE score after adjusting for age, sex, and years of education. Among those, SD and LGI in the parahippocampal and fusiform gyri had positive correlations with MMSE. For executive function, SD showed correlations in the left inferior frontal and orbitofrontal gyrus. Regarding language function, CT was associated with regions such as the superior temporal gyrus, while SD demonstrated correlations with the left supramarginal gyrus.

**Conclusion:**

The results indicate that certain changes in cortical shape features are associated with particular cognitive function scores. Surface-based morphometric features of SD and LGI provided complementary results to CT analyses. Region-specific changes in SD and LGI could be useful imaging markers to predict cognitive decline in AD patients.

## 1 Introduction

Alzheimer’s disease (AD) is the most prevalent form of dementia, primarily associated with advancing age, with an estimated 57 million cases worldwide as of 2019 (Nichols et al., 2022). AD is characterized by an acquired decline in cognitive abilities across multiple domains, leading to impairment in daily activities (Arvanitakis et al., 2019). It is also a major contributor to the loss of disability-adjusted life-years (DALYs) in patients over 75 years old (Vos et al., 2020).

Brief cognitive screening questionnaires have a key role in the early diagnosis and assessment of AD (Ismail et al., 2010). One such test is the Mini-Mental State Examination (MMSE), a brief 30-point questionnaire with a 50-year history (Folstein et al., 1975), still widely used to assess cognitive impairment (Milne et al., 2008). The Seoul Neuropsychological Screening Battery-II (SNSB-II) is a comprehensive neuropsychological evaluation tool, that provides essential information on early cognitive decline by assessing multiple divisional cognitive function scores, such as language and frontal/executive functions (Ryu and Yang, 2023).

Structural MRI plays a major role in excluding alternative non-AD etiologies of dementia and assessing patterns of neurodegeneration (Jack et al., 2024). While MRI-detectable changes in brain morphology were previously thought to emerge predominantly in later stages of AD, recent evidence suggests that structural alterations may appear much earlier in the course of disease. In particular, early patterns of cortical atrophy and network dysfunction have been observed even before overt cognitive decline (Javed et al., 2025). Reduced cortical thickness (CT) has been widely studied as a surrogate marker of neuronal loss. For example, a well-established “AD signature” of region-specific cortical thinning which involve inferior and middle temporal gyri, temporal pole, frontal and parietal association cortices, precuneus, etc. has been identified in mild cognitive impairment (MCI) and early AD, correlating with disease severity (Dickerson et al., 2009; Möller et al., 2013). Such cortical thinning reflects the underlying pathology and is strongly associated with declines in memory and other cognitive functions in AD (Jack et al., 2010; Keith et al., 2023). However, CT alone may not capture all aspects of cortical structural changes in AD, which therefore promotes interest in additional surface-based morphometric measures.

Surface-based morphometry (SBM) offers distinct advantages over voxel-based morphometry (VBM), as it enables vertex-level analysis of cortical surface shape and folding (Goto et al., 2022). Additional surface-based morphometric features such as sulcal depth (SD) and the local gyrification index (LGI) are not easily captured by volumetric approaches and offer complementary insights into cortical architecture. SD measures the distance from the brain’s outer cortical surface (i.e., convex hull) to the deepest point of each sulcus, therefore effectively quantifies how “deep” or pronounced each cortical fold is (Lyu et al., 2018a). In contrast, LGI captures the degree of cortical folding in each region, typically defined as the ratio of the folded (inner) surface area to the outer surface area of the cortex (Luders et al., 2006). These metrics reflect aspects of cortical geometry and complexity that are not captured by thickness alone – being influenced by developmental cortical folding patterns and structural connectivity (Liu et al., 2012). By characterizing the shape and complexity of gyri and sulci, SD and LGI therefore serve as valuable complements to CT in assessing neurodegenerative changes on the brain’s surface.

Recent studies indicate that both SD and LGI are altered in AD, revealing characteristic region-specific patterns and links to cognition. Cortical sulci tend to widen and become shallower in AD, resulting in reduced SD compared to age-matched healthy brain (Liu et al., 2012). For example, it was reported that individuals with AD or MCI exhibit significantly lower SD and curvature than cognitively normal controls, with the most prominent differences observed in the temporal lobes (Im et al., 2008). Similarly, global measures of cortical folding are diminished: even at very early stages of AD, overall cortical gyrification is lower than in cognitively normal individuals and continues to decline as the disease progresses (Liu et al., 2012; Lebed et al., 2013). Importantly, these morphological changes of lower global gyrification and greater sulcal expansion are associated with cognitive impairment. Moreover, specific cognitive domains map onto regional SD and LGI changes: recent work has shown that poorer memory, language, and executive function in AD are associated with reduced gyrification or shallower sulci in key areas (e.g., inferior temporal and supramarginal gyri) independent of CT (Coleman et al., 2023). Furthermore, emerging evidence suggests that cortical folding geometry may influence not only cognitive outcomes but also the effectiveness of neuromodulatory interventions such as transcranial alternating current stimulation (tACS), as demonstrated in computational and experimental work (Cabrera-Álvarez et al., 2023). These findings imply that AD-related neurodegeneration is accompanied by an abnormal “unfolding” of the cortical surface, manifesting as altered SD and LGI in key brain regions.

Despite clinical findings, the conventional LGI approach is limited in ways that may hinder accurate quantification of cortical folding. First, it relies on fixed-size spherical patches on cortical surfaces, which may not adequately account for individual variability in brain size or folding patterns (Lyu et al., 2018b). For instance, even if identical shapes are presented at different scales, their computed gyrification can differ despite the LGI intending to quantify the ratio of cortical to outer surface area, which should be scale-invariant. Second, the conventional local patch follows a simple, fixed shape that likely spans both gyral and sulcal regions even when these belong to distinct sulcal structures. As pointed out in Power et al. (2011) and Wig et al. (2014), human brain functions tend to be locally homogeneous within a sulcus or gyrus, but the conventional approach is limited in its ability to capture such functional and anatomical specificity. Third, traditional approaches focus solely on the LGI, which measures the ratio of cortical to outer surface area. However, this metric cannot distinguish between changes in SD and width, potentially overlooking important morphological variations. To address these limitations, we adopted an improved LGI computation method that uses shape-adaptive patches, enhancing spatial specificity by including only anatomically relevant regions around each vertex and offering improved methodological reliability (Lyu et al., 2018b). Płonka et al. (2020) have further demonstrated improved sensitivity in localizing group-level differences in cortical folding compared to the conventional method. This approach has been then applied in various contexts, including neurodevelopmental studies (Lyu et al., 2018b) and investigations of neurological disorders such as Huntington’s disease (Stoebner et al., 2023) and autism spectrum disorders (Zoltowski et al., 2021; Lucibello et al., 2022).

While each of the three morphometric measures (CT, SD, and LGI) has been studied individually, few studies have examined them collectively in relation to cognitive outcomes in AD. This leaves a gap in our understanding of how these imaging markers jointly contribute to cognitive decline. In this context, we hypothesized that cortical folding features including SD and LGI, alongside CT would show distinct associations with both global and domain-specific cognitive scores in patients with AD. A simultaneous analysis of these morphometric features may improve our understanding of how regional brain structure relates to cognitive function and would provide valuable support for decision-making and prognoses in clinical settings.

Therefore, this study aimed to investigate the relationship between three cortical shape features—CT, SD, and LGI—and cognitive performance in AD, thereby providing a multidimensional cortical profile for AD-related cognitive decline.

## 2 Materials and methods

### 2.1 Patient population

**FIGURE 1:**
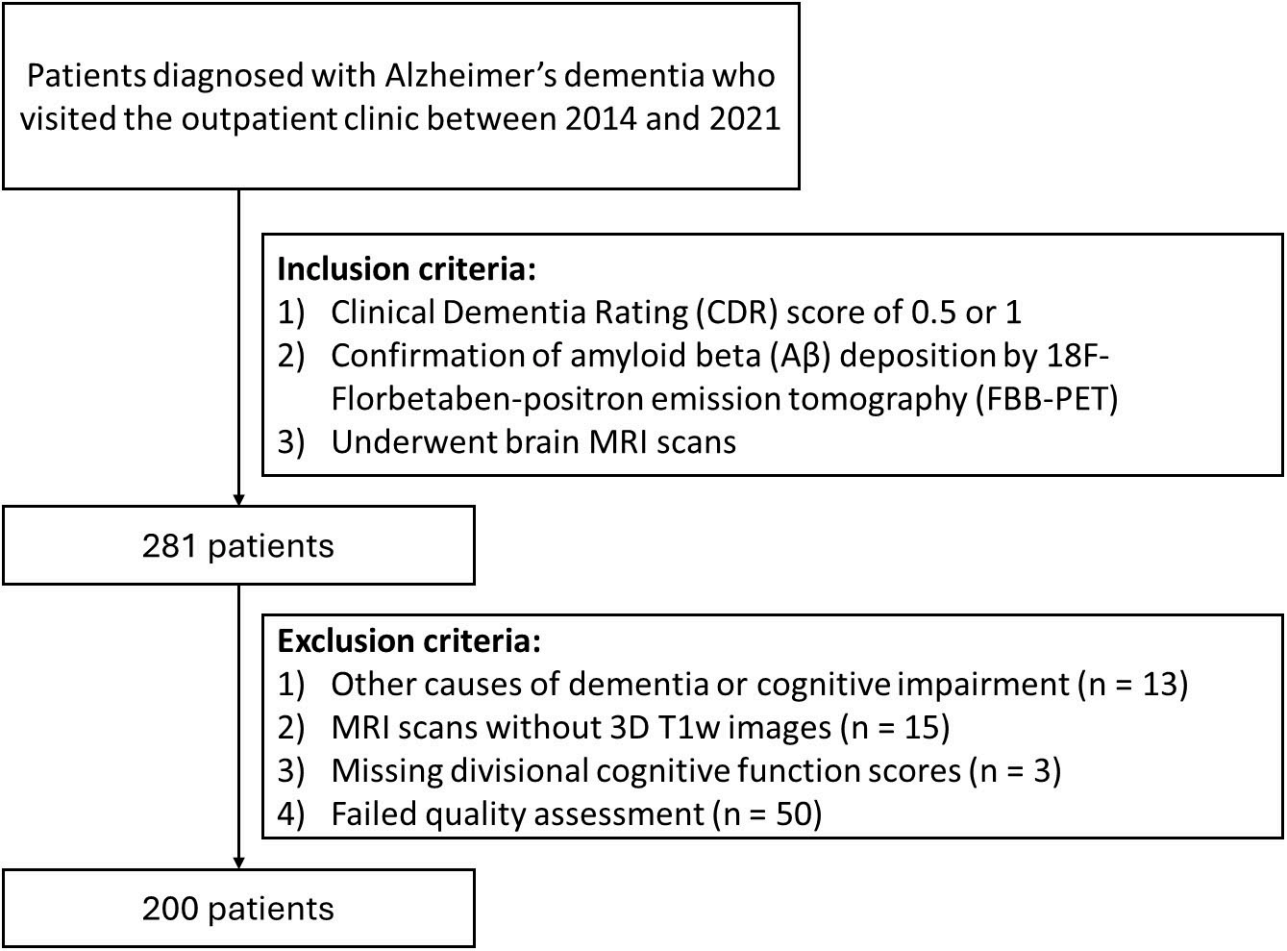
Flowchart for patient inclusion.

This study was approved by the Institutional Review Board, and the requirement for written informed consent was waived due to the retrospective design of the study and use of de-identified data. A total of 281 consecutive patients with AD who visited the dementia clinic at Severance Hospital between 2014 and 2021 and had a Clinical Dementia Rating (CDR) score of 0.5 or 1 were included in the study. The diagnosis of AD was established according to the 2011 National Institute on Aging–Alzheimer’s Association (NIA-AA) criteria. Diagnoses were made through a consensus panel of neurologists (Y.-G.L. and B.Y.), with confirmation of amyloid beta (Aβ) deposition via 18F-florbetaben-positron emission tomography (FBB-PET). The following criteria were used for exclusion: (1) diagnoses of other types of dementia, including frontotemporal dementia, dementia with Lewy bodies, corticobasal degeneration, and progressive supranuclear palsy, (2) cognitive impairment caused by medications, (3) the presence of other potential causes of cognitive impairment, such as epilepsy, psychiatric disorder, or structural brain lesions, and (4) insufficient or inadequate MRI scans or cognitive function scores. Figure 1 shows a flowchart for patient enrollment.

### 2.2 Clinical data

The demographic and clinical data were obtained from patient medical records, which included age, sex, level of education, and presence of comorbidities of hypertension, type 2 diabetes, dyslipidemia, and history of cerebrovascular accident (CVA). All patients underwent a comprehensive neuropsychological assessment, which included the MMSE and SNSB-II (Ryu and Yang, 2023). Regarding the SNSB-II scores, each test score was first transformed into a *z*-score to standardize the results, allowing for the composite score to reflect an individual’s cognitive performance relative to the study population. The language function score was defined as *z*-scores of the Korean version of the Boston Naming Test (BNT) (Kim and Na, 1999). The executive function score is calculated by the summation of *z*-scores of the Controlled Oral Word Association Test (COWAT) and Color Word Stroop Test (CWST) scores. The attention score is calculated by the summation of *z*-scores of the digit span forward and digit span backward scores. The visuospatial function score is determined by the *z*-score of the Rey Complex Figure Test (RCFT) score. Memory function is assessed by summing the *z*-scores of the Seoul Verbal Learning Test (SVLT) immediate recall, SVLT delayed recall, SVLT recognition, RCFT copying, RCFT immediate recall, and RCFT delayed recall scores.

### 2.3 MRI and PET acquisition

Structural MRI scans were performed using the 3-T system (Ingenia CX, Philips Healthcare) equipped with a 32-channel head coil, with a 3D magnetization-prepared rapid acquisition with gradient echo (MPRAGE) sequence utilized for T1-weighted images (T1w). Supplementary Data shows the details of the MRI acquisition parameters.

18F-Florbetaben-positron emission tomography scans were performed using the Discovery 600 system (GE Healthcare). Participants received an intravenous injection of 300 MBq (8 mCi) of FBB. Image acquisition began 90 min after injection and continued for 20 min. PET images were reconstructed using the ordered subset expectation maximization (OSEM) algorithm with 4 iterations and 32 subsets. A Gaussian filter with a 4-mm full width at half maximum (FWHM) was applied to the reconstructed images. Then, Aβ positivity was determined using a global FBB standardized uptake value ratio (SUVR) cutoff of 1.478.

The acquired MR images were processed via FreeSurfer v7.4.1, including N4 bias correction, skull-stripping, tissue segmentation, intensity normalization, and cortical surface reconstruction. The reconstructed surfaces were spherically mapped and registered to *fsaverage* template surface (Fischl et al., 1999) for shape correspondence using the hierarchical spherical deformation method that reduces registration distortion (Lyu et al., 2019). After registration, each surface was resampled into 163,842 vertices using the icosahedral regular grid to establish shape correspondence across subjects. It is important to note that the resampled surface was only used for statistical shape analysis. To prevent any potential information loss associated with the resampling process, other surface-related processing was conducted on the originally reconstructed surface.

### 2.4 Shape feature extraction

**FIGURE 2:**
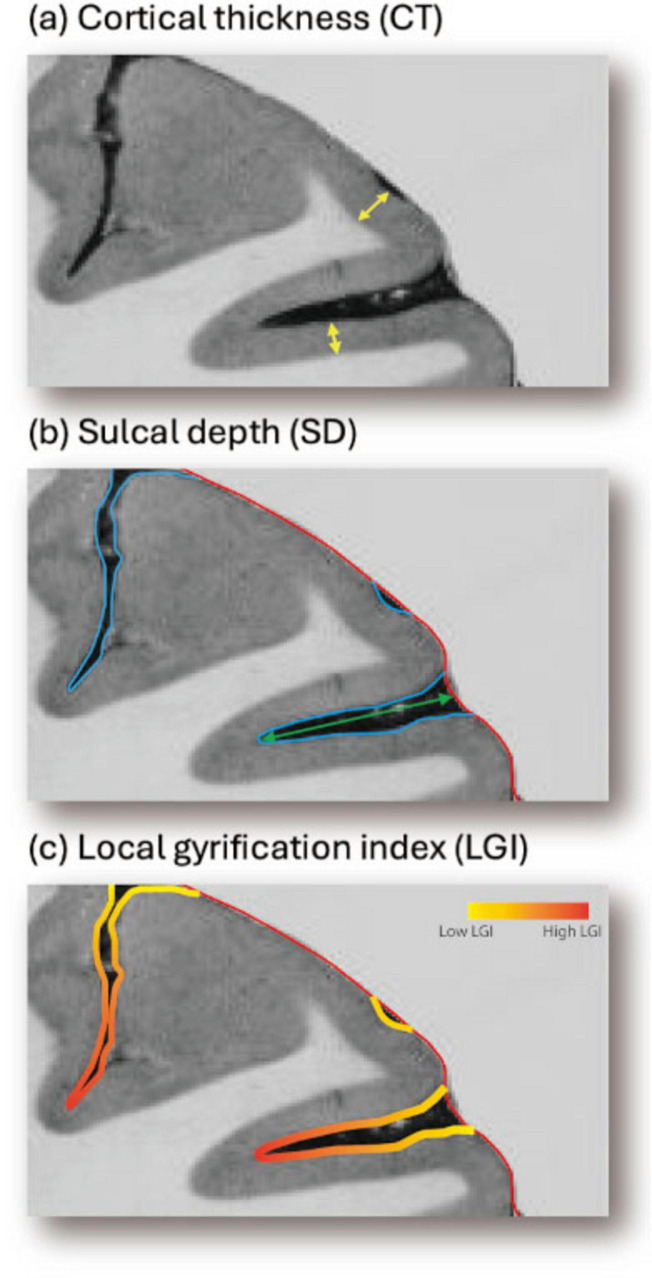
Overview of the methodology for measuring **(a)** CT, **(b)** SD, and **(c)** LGI on the cerebral surface. CT is measured as the minimal distance between corresponding point on the pial and white matter surface (yellow bar). SD is measured as the Laplacian trajectory between CHS (red contour) and the pial surface (blue contour). LGI is then measured as the ratio between the pial (blue) and CHS (red) surface area. The color gradients for LGI indicates the regions expected to have higher values (shown in orange) and lower values (shown in yellow). CHS, cerebral hull surface; CT, cortical thickness; LGI, local gyrification index; SD, sulcal depth

Three cortical shape features—CT, SD, and LGI—were extracted from the reconstructed cortical surface for analysis. The measurement of CT, which refers to the width of the cortical gray matter, was calculated using the FreeSurfer (Fischl, 2012). The cerebral hull surface (CHS), which is the virtual outer contour of the cerebral cortex, was used as the reference for the measurement of SD (Lyu et al., 2018a) and LGI (Lyu et al., 2018b). SD is defined as the Laplacian trajectory between CHS and pial surface, while LGI represents the ratio between CHS area and pial surface area at each point on the inner contour using a shape-adaptive kernel. This definition of SD is conceptually distinct from FreeSurfer’s SD (also referred to as average convexity), which is computed as the displacement along the inflation trajectory from the cortical surface to the inflated surface rather than CHS. CT and SD were then smoothed over the cortical surface using a Gaussian kernel with a FWHM of 6 mm in FreeSurfer for denoising and improving spatial consistency. Figure 2 provides an overview of the methodology for measuring CT, SD, and LGI.

### 2.5 Statistical analysis

A comprehensive whole-brain vertex-wise analysis was conducted using a general linear model (GLM) in SurfStat (Worsley et al., 2009), a toolbox designed for cortical SBM analysis, in MATLAB v.2021a.

Statistical inference was performed using random field theory (RFT) to control the family-wise error (FWE) rate from multiple comparisons. Cluster-level significance was assessed at FWE-corrected threshold of *p* < 0.05, using cluster-forming height threshold of uncorrected *p* < 0.01.

In order to examine the association between MMSE scores and each of three cortical shape features while controlling for year of education, age and sex, the following model was specified: (*shape feature*) = β_0_ + β_1_ (*MMSE*) + β_2_ (*year of education*) + β_3_ (*age*) + β_4_ (*sex*) + ε. The null hypothesis of *H*_0_: β_1_ = 0 was tested, and vertex-wise *t* values and standardized β were computed to evaluate the strength and spatial extent of the observed effects. For domain-specific analysis, the model was extended by including divisional cognitive function scores as an additional predictor: (*shape feature*) = β_0_ + β_1_ (*divisional cognitive function score*) + β_2_ (*MMSE*) + β_3_ (*year of education*) + β_4_ (*age*) + β_5_ (*sex*) + ε. The corresponding null hypothesis *H*_0_: β_1_ = 0 was tested, and corresponding *t* values and standardized β were computed.

## 3 Results

### 3.1 Patient characteristics

A total of 200 patients were enrolled in this study, excluding those with other causes of dementia or cognitive impairment (*n* = 13) and those without 3D T1w (*n* = 15). Additionally, patients with missing cognitive scores (*n* = 3) and those with T1w scans that failed quality assessment (*n* = 50) were excluded (Figure 1). Quality assessment failures included cropped or noisy scans, inaccurate automated tissue segmentation, and mesh artifacts in the reconstructed cortical surfaces.

A total of 62.5% (*n* = 125) of patients were female, with a mean age ± standard deviation (Stdev) of 73.7 ± 7.1 years (range: 55.5–87.1). The mean ± Stdev duration of education was 10.4 ± 4.7 years (range: 0.5–18) and the mean ± Stdev MMSE score was 23.8 ± 3.0 (range: 15–30). Hypertension was the most common comorbidity, affecting 56.0% (*n* = 112) of patients, followed by dyslipidemia (24.0%, *n* = 48) and type 2 diabetes (20.0%, *n* = 40). Table 1 summarizes the baseline characteristics of the cohort.

**TABLE 1:** Patient characteristics.

Characteristics	AD (*n* = 200)
Female, *n* (%)	125 (62.5)
Age, mean ± Stdev	73.7 ± 7.1
Education, mean ± Stdev	10.4 ± 4.7
MMSE score, mean ± Stdev	23.8 ± 3.0
Hypertension, *n* (%)	112 (56.0)
Type 2 diabetes, *n* (%)	40 (20.0)
Dyslipidemia, *n* (%)	48 (24.0)
History of CVA, *n* (%)	12 (6.0)

CVA, cerebrovascular accident; MMSE, Mini-Mental State Examination; Stdev, standard deviation.

### 3.2 Cortical shape features and MMSE

**FIGURE 3:**
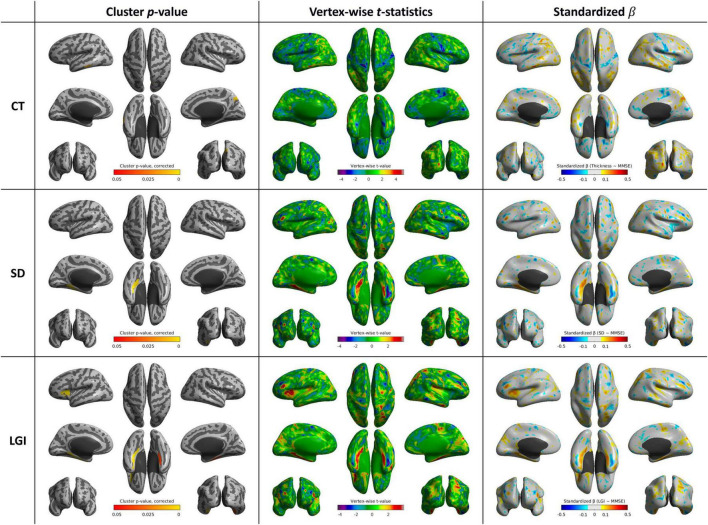
Whole-brain statistical maps showing associations between MMSE scores and cortical morphometry: CT (top), SD (middle), and LGI (bottom). From left to right: corrected cluster-wise *p* values, vertex-wise *t* values, and standardized β coefficients. CT, cortical thickness; LGI, local gyrification index; SD, sulcal depth

After controlling age, sex, and year of education, CT was positively correlated with MMSE score in the right precuneus (*p* = 0.006) and left inferior temporal gyrus (*p* = 0.013). SD was positively correlated with the MMSE score in the left parahippocampal and fusiform gyri (*p* = 0.002). LGI was positively correlated with MMSE score in left parahippocampal and fusiform gyri (*p* < 0.001), left inferior frontal gyrus (*p* < 0.001), and right parahippocampal and fusiform gyri (*p* = 0.035) (Table 2 and Figure 3).

**TABLE 2:** List of cortical clusters showing significant associations between cortical shape features and MMSE scores.

Cortical feature	Cluster	Cluster size (vertices)	Peak *t*-value	Corrected *p*-value (FWE)
CT	Right precuneus	561	3.55	0.006
CT	Left inferior temporal gyrus	284	4.69	0.013
SD	Left parahippocampal, fusiform, and lingual gyri	1,489	3.98	0.002
LGI	Left parahippocampal, fusiform, and lingual gyri	1,718	3.95	<0.001
LGI	Left inferior frontal gyrus	1,542	3.92	<0.001
LGI	Right parahippocampal and fusiform gyri	1,058	3.56	0.035

CT, cortical thickness; FWE, family-wise error; LGI, local gyrification index; MMSE, Mini-Mental State Examination; SD, sulcal depth.

### 3.3 Cortical shape features and executive function score

**FIGURE 4:**
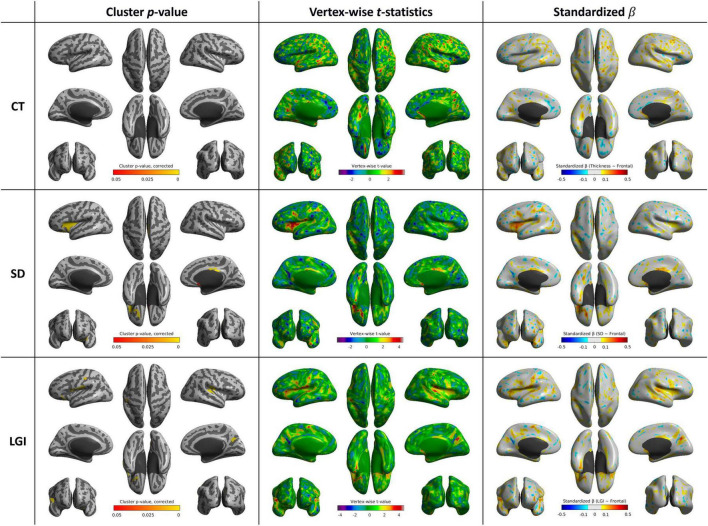
Whole-brain statistical maps showing associations between executive function scores and cortical morphometry: CT (top), SD (middle), and LGI (bottom). From left to right: corrected cluster-wise *p* values, vertex-wise *t* values, and standardized β coefficients. CT, cortical thickness; LGI, local gyrification index; SD, sulcal depth

After controlling age, sex, year of education, and MMSE score, no brain region showed a significant positive correlation between CT and executive function scores. SD was positively correlated with executive function scores in the left inferior frontal gyrus (*p* < 0.001), orbitofrontal cortex (*p* < 0.001), right middle cingulate gyrus (*p* < 0.001), and right subcallosal area (*p* = 0.044). LGI was positively correlated with executive function scores in the bilateral postcentral and supramarginal gyri (*p* < 0.001 and *p* = 0.002, respectively), right superior temporal gyrus (*p* = 0.002), right precuneus (*p* = 0.002), and left orbitofrontal cortex (*p* = 0.001) (Table 3 and Figure 4).

**TABLE 3:** List of cortical clusters showing significant associations between cortical shape features and executive function scores.

Cortical feature	Cluster	Cluster size (vertices)	Peak *t*-value	Corrected *p*-value (FWE)
SD	Left inferior frontal gyrus	3,294	4.51	<0.001
SD	Left orbitofrontal cortex	626	4.39	<0.001
SD	Right middle cingulate gyrus	603	3.38	<0.001
SD	Right subcallosal area	92	4.13	0.044
LGI	Left postcentral and supramarginal gyri	2,444	3.99	<0.001
LGI	Right postcentral and supramarginal gyri	1,319	4.51	0.002
LGI	Right superior temporal gyrus	1,024	4.02	0.002
LGI	Right precuneus	463	4.69	0.002
LGI	Left orbitofrontal cortex	438	3.59	0.001

CT, cortical thickness; FWE, family-wise error; LGI, local gyrification index; SD, sulcal depth.

### 3.4 Cortical shape features and language function score

**FIGURE 5:**
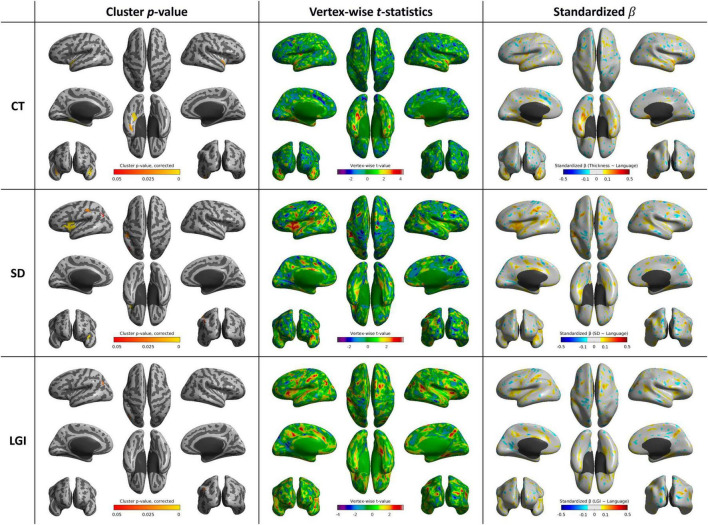
Whole-brain statistical maps showing associations between language function scores and cortical morphometry: CT (top), SD (middle), and LGI (bottom). From left to right: corrected cluster-wise *p* values, vertex-wise *t* values, and standardized β coefficients. CT, cortical thickness; LGI, local gyrification index; SD, sulcal depth

Cortical thickness was positively correlated with language function score in the left parahippocampal and fusiform gyri (*p* < 0.001), bilateral superior temporal gyrus (*p* < 0.001 and *p* = 0.015, respectively), and left fusiform and inferior temporal gyri (*p* = 0.017). SD was positively correlated with language function scores in the left inferior frontal gyrus (*p* < 0.001), left supramarginal and postcentral gyri (*p* = 0.021), and left inferior parietal gyrus (*p* = 0.046). LGI was associated with language function scores in the left inferior parietal gyrus (*p* = 0.028) (Table 4 and Figure 5).

**TABLE 4:** List of cortical clusters showing significant associations between cortical shape features and language function scores.

Cortical feature	Cluster	Cluster size (vertices)	Peak *t*-value	Corrected *p*-value (FWE)
CT	Left parahippocampal and fusiform gyri	865	3.65	<0.001
CT	Left superior temporal gyrus	773	3.63	<0.001
CT	Right superior temporal gyrus	584	4.02	0.015
CT	Left fusiform and inferior temporal gyri	513	4.40	0.017
SD	Left inferior frontal gyrus	2,449	3.22	<0.001
SD	Left supramarginal and postcentral gyri	1,063	3.66	0.021
SD	Left inferior parietal gyrus	266	3.26	0.046
LGI	Left inferior parietal gyrus	282	3.51	0.028

CT, cortical thickness; FWE, family-wise error; LGI, local gyrification index; SD, sulcal depth.

### 3.5 Cortical shape features and attention function score

**FIGURE 6:**
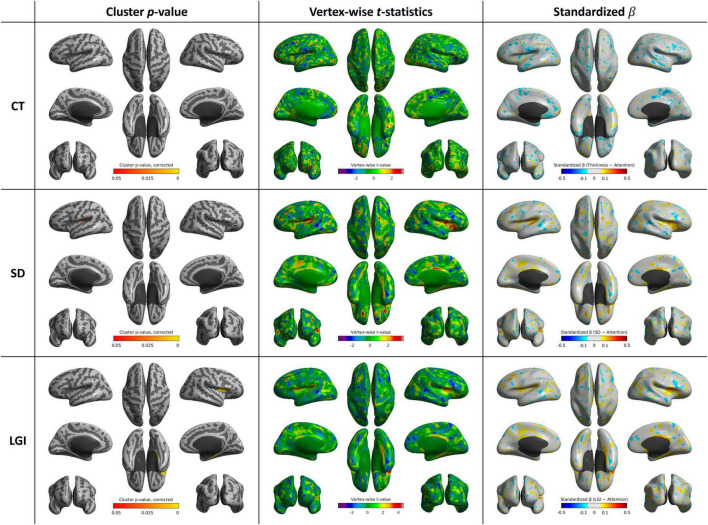
Whole-brain statistical maps showing associations between attention function scores and cortical morphometry: CT (top), SD (middle), and LGI (bottom). From left to right: corrected cluster-wise *p* values, vertex-wise *t*-statistics, and standardized β coefficients. CT, cortical thickness; SD, sulcal depth; LGI, local gyrification index.

No brain region showed a significant positive correlation between CT and attention function scores. SD was positively correlated with attention function scores in the left postcentral and supramarginal gyri (*p* = 0.025). LGI was associated with attention function scores in the right inferior frontal gyrus (*p* < 0.001) and right entorhinal and parahippocampal gyri (*p* < 0.001) (Supplementary Table 1 and Figure 6).

### 3.6 Cortical shape features and memory function score

**FIGURE 7:**
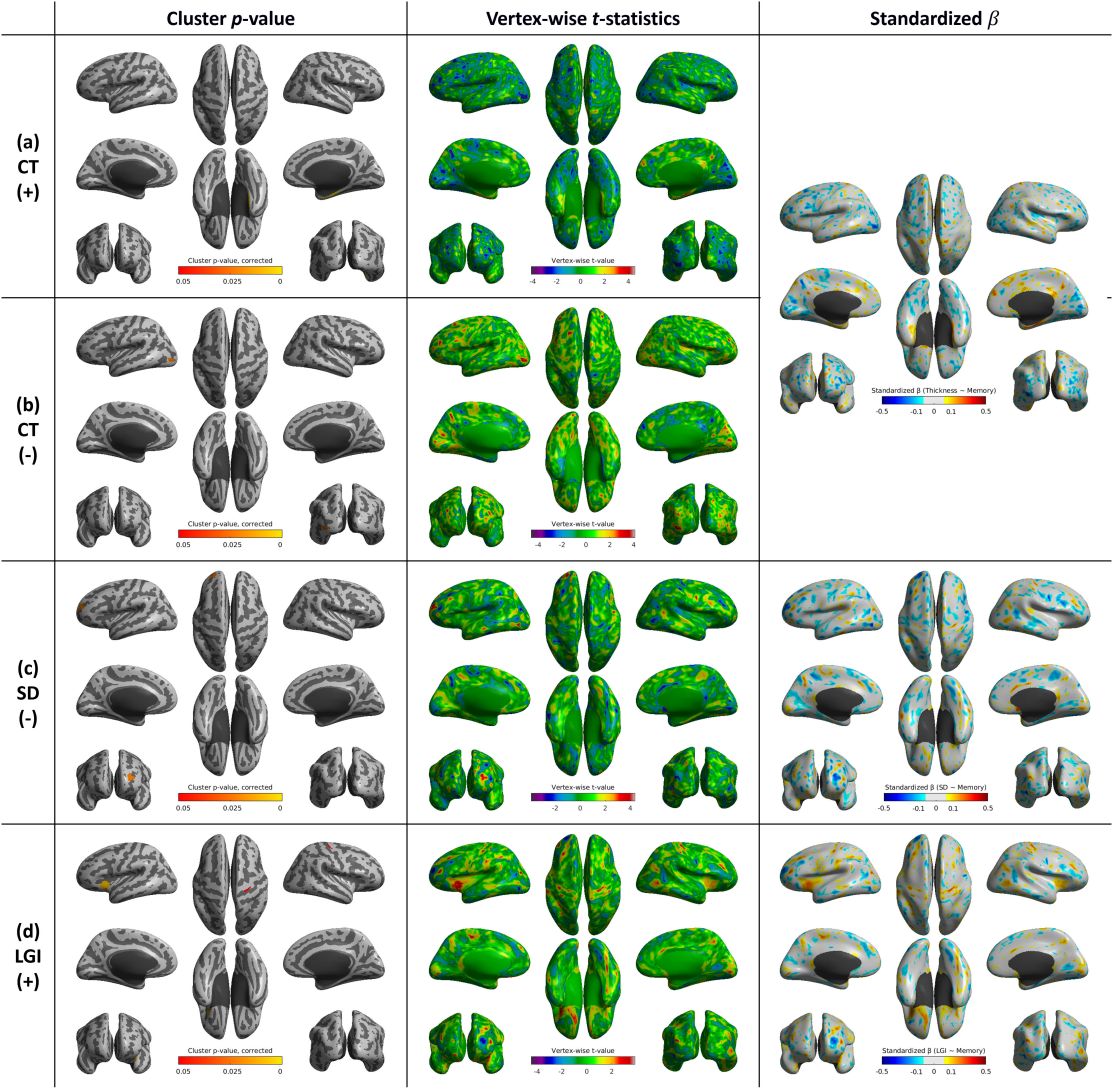
Whole-brain statistical maps showing associations between memory function scores and cortical morphometry: **(a)** CT (positive correlation), **(b)** CT (negative correlation), **(c)** SD (negative correlation), and **(d)** LGI (positive correlation). From left to right: corrected cluster-wise *p* values, vertex-wise *t*-statistics, and standardized β coefficients. CT, cortical thickness; SD, sulcal depth; LGI, local gyrification index.

We observed mixed positive and negative correlations between cortical shape features and memory function scores in specific brain regions. CT showed a significant positive correlation memory function scores in the right parahippocampal gyrus (*p* < 0.001), while there was also negative correlation with memory function scores in the left lateral occipital gyrus (*p* = 0.024). SD was negatively associated with memory function scores in the left superior frontal gyrus (*p* = 0.023). Additionally, LGI demonstrated a significant positive correlation with memory function scores in the left insula (*p* = 0.008) and right precentral gyrus (*p* = 0.049) (Supplementary Table 2 and Figure 7).

### 3.7 Cortical shape features and visuospatial function score

**FIGURE 8:**
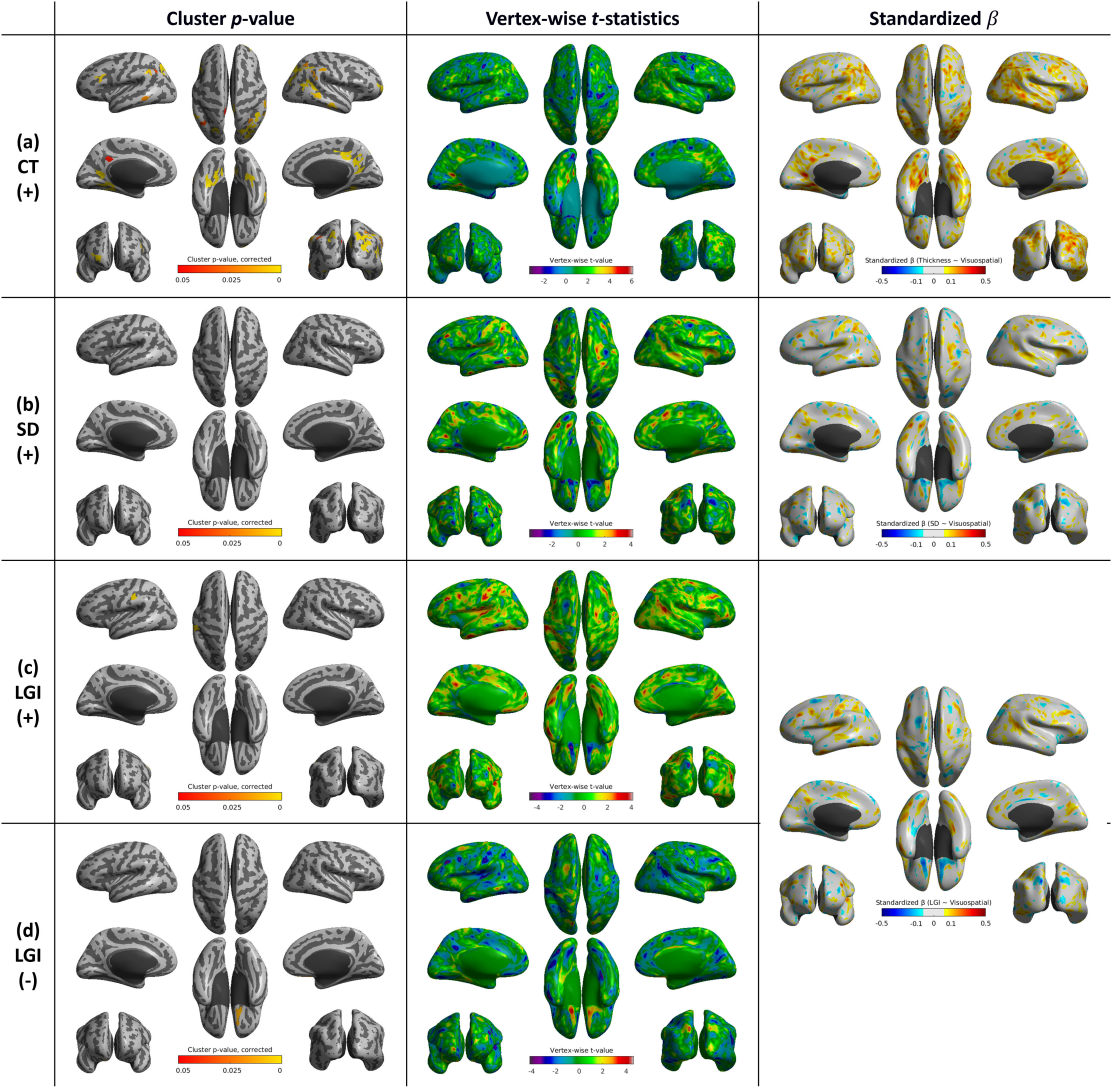
Whole-brain statistical maps showing associations between visuospatial function scores and cortical morphometry: **(a)** CT, **(b)** SD, **(c)** LGI (positive correlation), and **(d)** LGI (negative correlation). From left to right: corrected cluster-wise *p* values, vertex-wise *t*-statistics, and standardized β coefficients. CT, cortical thickness; SD, sulcal depth; LGI, local gyrification index.

Cortical thickness exhibited positive correlations with visuospatial function scores in extensive areas of brain regions, especially including bilateral occipital and temporal lobes, and supramarginal and angular gyri. LGI showed significant positive correlation in the left supramarginal and postcentral gyri (*p* = 0.004), while displaying a negative correlation in the right orbitofrontal cortex (*p* = 0.013) (Supplementary Table 3 and Figure 8).

## 4 Discussion

### 4.1 Summary of main findings

In this study, we investigated the regional associations between three cortical shape features—CT, SD, and LGI—and cognitive performance scores measured by MMSE and multiple divisional cognitive function scores in patients with AD. By controlling for age, sex, and year of education, we were able to identify specific regions where cortical morphology correlates with cognitive performance scores. Our findings reveal that MMSE scores were positively correlated with CT in the left inferior temporal gyrus and right precuneus, SD in the left parahippocampal and fusiform gyri, and LGI in the bilateral parahippocampal gyri, left fusiform and inferior frontal gyri, and right parahippocampal gyrus. For executive function scores, CT showed no significant association, while SD was correlated with the left inferior frontal gyrus, orbitofrontal cortex, right middle cingulate gyrus, and subcallosal area. LGI was positively associated with executive scores in the bilateral postcentral gyri, left orbitofrontal cortex, right precuneus, and right superior temporal gyrus. Language function scores were positively correlated with CT in the left parahippocampal and fusiform gyri and bilateral superior temporal gyri, and with SD in the left inferior frontal, supramarginal, and inferior parietal gyri. LGI was associated with language performance only in a small region of the left inferior parietal gyrus. For attention, CT showed no significant correlation, whereas SD was positively associated with the left postcentral and supramarginal gyri, and LGI with the right inferior frontal gyrus. Memory function showed both positive and negative correlations: CT was positively associated with the right parahippocampal gyrus and negatively with the left lateral occipital gyrus; SD showed a negative association with the left superior frontal gyrus; and LGI was positively correlated with the left insula and right precentral gyri. Visuospatial function scores were associated with CT in widespread regions, including the bilateral occipital and temporal lobes and the supramarginal and angular gyri. LGI showed a positive correlation in the left supramarginal gyrus and a negative correlation in the right orbitofrontal cortex. Overall, these findings highlight the regional specificity of cortical shape features in relation to distinct cognitive functions in AD.

### 4.2 MMSE and global cognitive function

The MMSE comprises questionnaires for the concise evaluation of multiple cognitive domains, including attention, orientation, memory, registration, recall, calculation, and language function (Folstein et al., 1975). This broad scope may explain why different cortical shape features of multiple brain regions showed a significant correlation with the MMSE score. In particular, the involvement of both posterior and parietal regions suggests a network that supports integrated visuospatial processing and cognitive function (Kravitz et al., 2011). SD and LGI analyses showed positive correlations with MMSE scores in various regions linked to memory formation and retrieval, language processing, somatosensory functions, and motor control, indicating that overall cognitive abilities are influenced by both CT and sulcal folding complexity. Interestingly, the spatial extent of CT–MMSE associations was relatively limited in our sample, possibly due to the restricted variance in cognition decline across the mild-to-moderate AD cohort and the stringent surface-based statistical correction applied. Notably, LGI demonstrated broader and more robust associations with MMSE, potentially reflecting its sensitivity to both SD and width as markers of advanced cortical remodeling.

### 4.3 Executive function and frontal morphology

Executive functions, also sometimes referred to as frontal functions (Fuster, 2000), are cognitive processes that are key to goal-directed behavior, planning, decision-making, and problem-solving (Alvarez and Emory, 2006). The prefrontal cortex, in particular, is known to play a key role in reward-related decision-making and inhibitory control, aligning with its correlation to executive function (Friedman and Robbins, 2022). SD, rather than CT, in the lateral prefrontal cortex was shown to be a significant indicator of working memory performance (Yao et al., 2023). Similarly, SD—but not CT—in the inferior frontal gyrus and orbitofrontal cortex was associated with our executive function scores. This suggests that alterations in the sulcal structure of these regions could be linked to decline in executive function performance in patients with AD. In addition, LGI in the postcentral gyri, precuneus, and superior temporal gyrus—regions implicated in sensorimotor integration and attentional control—showed significant associations with executive performance, which is possible reflection of the role of multimodal networks in supporting task execution.

### 4.4 Language and visuospatial correlates

Loss of language function occurs in a significant proportion of AD patients (Henry et al., 2004) and is a major contributor to the deterioration of their daily abilities, making it a key target for early therapeutic intervention (Verma and Howard, 2012). In our study, language performance was evaluated using BNT, which is widely used for the assessment of naming deficits in AD. Our analysis shows that language function scores are associated with cortical features in the superior temporal and supramarginal gyri, regions well-known for their role in speech and language processing (Friederici, 2011). This also aligns with previous research that showed that overall performance on the BNT is associated with a left hemispheric network including the middle and superior temporal gyrus and extending into the inferior parietal cortex (Baldo et al., 2013). The fusiform gyrus, anatomically linked to object recognition processes (Weiner and Zilles, 2016), may structurally support naming abilities as measured by the BNT; however, this association should be interpreted in light of the structural nature of the imaging data rather than direct functional inference. However, the minimal LGI association limited to the left inferior parietal gyrus suggests that cortical folding complexity may be less involved in language performance, at least as assessed by naming tasks. Additional domain-specific tests targeting syntax or semantic fluency may be needed to better assess LGI sensitivity.

The association between CT and visuospatial function across widespread parieto-occipital regions supports the involvement of the dorsal visual pathway, which may contribute to the execution of visuospatial tasks, consistent with findings from previous studies (Kang et al., 2019). Overall, our results suggest that distinct alterations in cortical shape features are selectively associated with specific cognitive domains in patients with AD.

### 4.5 Clinical implications and interpretability of LGI

Cortical thinning, particularly in temporal and parietal association cortices, was most consistently associated with memory and language performance, in line with previous work identifying the “AD signature” pattern of cortical atrophy. SD reductions were observed in similar regions, but also extended to frontal areas, suggesting that SD may detect early geometric alterations related to neurodegeneration. LGI changes were more regionally restricted but nonetheless informative: lower gyrification in the supramarginal and insular cortices correlated with poorer executive and language performance, consistent with recent studies linking cortical complexity to cognitive resilience in AD.

Importantly, our findings demonstrate that CT, SD, and LGI are non-redundant features capturing complementary aspects of cortical structure. In several regions, cognitive outcomes were predicted more accurately by a combination of CT, SD, and LGI than by CT alone. Among the three features, LGI demonstrated relatively broader and more robust associations across domains—particularly in MMSE, executive, and visuospatial scores—highlighting its potential as a sensitive marker of cortical remodeling. While CT remains the most sensitive index of global cortical atrophy, SD and LGI provide additional geometrical and topographical insights that may capture subtle cortical remodeling and support prediction of cognitive decline. This may be because LGI not only considers simple SD but also includes the ratio of the pial surface to the CHS, which is influenced by both the depth and width of the sulcus (Schaer et al., 2008). This allows for a more detailed reflection of cortical morphology compared to SD.

A primary strength of this study is the application of cutting-edge techniques to analyze the cortical shape features, with a particular focus on evaluating the usefulness of LGI. This study employed techniques that adapt to individual brain size and the structural pattern of local cortical folding, accounting for individual differences and providing more reproducible results (Lyu et al., 2018b). By employing a shape-adaptive LGI approach, we improved anatomical specificity and reduced variability, supporting its potential as a practical biomarker. Through such reliable analysis of cortical shape features, future research might not only analyze associations but also predict and measure an AD patient’s cognitive function to some extent.

### 4.6 Limitations and future directions

Some limitations warrant caution during the interpretation of this study’s findings. This retrospective study was conducted at a single center, which included a relatively small number of patients. This was also an exploratory study and did not account for patient-specific medical history or other socioeconomic factors (Sattler et al., 2012) that may have influenced the global and domain-specific cognitive scores. MMSE and domain-specific cognitive scores are also affected by measurement errors and a patient’s overall health condition, potentially leading to variability in results (Clark et al., 1999).

Although we focused on assessing the main effects of each cortical morphometric feature, it would be beneficial to examine potential interaction effects (e.g., CT × SD, CT × LGI) to identify complex structural profiles. Future research should explore these interactions to elucidate whether combinations of cortical metrics can improve the prediction of cognitive decline trajectories.

Another limitation is the unavailability of continuous SUVR data from the FBB-PET scans, which precluded the incorporation of amyloid-β burden as a covariate or moderator in the surface-based analyses. Future longitudinal studies are needed to clarify the relative contributions of Aβ pathology and downstream neurodegeneration to cortical morphological changes.

Finally, while our study highlights regional specificity of morphometric features, the cross-sectional design limits inference on temporal progression. Future longitudinal studies are needed to evaluate the predictive value of sulcal morphometry features for cognitive progression in AD and to explore their utility in tracking disease-modifying treatment responses.

## 5 Conclusion

Our findings demonstrate that CT, SD, and LGI each show distinct and region-specific associations with global and domain-specific cognitive performance in AD patients. Surface-based morphometric features of SD and LGI provided complementary results to CT analyses. While CT remains a well-established marker of cortical atrophy, SD and LGI offer additional geometrical and topographical insights into sulcal remodeling and folding complexity that are not captured by CT alone. These metrics were particularly informative for functions such as executive processing and visuospatial skills, where CT alone showed limited associations. This multidimensional profiling offers a more precise characterization of imaging biomarkers in clinical research and may guide individualized intervention strategies. Further research with more diverse cohorts is necessary to generalize and extend these results.

## Data Availability

The raw data supporting the conclusions of this article will be made available by the authors, without undue reservation.

## References

[B1] Alvarez J. A. Emory E. (2006). Executive function and the frontal lobes: A meta-analytic review. *Neuropsychol. Rev.* 16 17–42. 10.1007/s11065-006-9002-x 16794878

[B2] Arvanitakis Z. Shah R. C. Bennett D. A. (2019). Diagnosis and management of dementia: Review. *JAMA* 322 1589–1599. 10.1001/jama.2019.4782 31638686 PMC7462122

[B3] Baldo J. V. Arévalo A. Patterson J. P. Dronkers N. F. (2013). Grey and white matter correlates of picture naming: Evidence from a voxel-based lesion analysis of the Boston Naming Test. *Cortex* 49 658–667. 10.1016/j.cortex.2012.03.001 22482693 PMC3613759

[B4] Cabrera-Álvarez J. Sánchez-Claros J. Carrasco-Gómez M. del Cerro-León A. Gómez-Ariza C. J. Maestú F. (2023). Understanding the effects of cortical gyrification in tACS: Insights from experiments and computational models. *Front. Neurosci.* 17:1223950. 10.3389/fnins.2023.1223950 37655010 PMC10467425

[B5] Clark C. M. Sheppard L. Fillenbaum G. G. Galasko D. Morris J. C. Koss E. (1999). Variability in annual mini-mental state examination score in patients with probable Alzheimer disease: A clinical perspective of data from the consortium to establish a registry for Alzheimer’s disease. *Arch. Neurol.* 56 857–862. 10.1001/archneur.56.7.857 10404988

[B6] Coleman M. M. Keith C. M. Wilhelmsen K. Mehta R. I. Vieira Ligo, Teixeira C. (2023). Surface-based correlates of cognition along the Alzheimer’s continuum in a memory clinic population. *Front. Neurol.* 14:1214083. 10.3389/fneur.2023.1214083 37731852 PMC10508059

[B7] Dickerson B. C. Bakkour A. Salat D. H. Feczko E. Pacheco J. Greve D. N. (2009). The cortical signature of Alzheimer’s disease: Regionally specific cortical thinning relates to symptom severity in very mild to mild AD dementia and is detectable in asymptomatic amyloid-positive individuals. *Cereb Cortex* 19 497–510. 10.1093/cercor/bhn113 18632739 PMC2638813

[B8] Fischl B. (2012). FreeSurfer. *Neuroimage* 62 774–781. 10.1016/j.neuroimage.2012.01.021 22248573 PMC3685476

[B9] Fischl B. Sereno M. I. Tootell R. B. H. Dale A. M. (1999). High-resolution intersubject averaging and a coordinate system for the cortical surface. *Hum. Brain Mapp.* 8 272–284. 10.1002/(sici)1097-0193(1999)8:4<272::aid-hbm10>3.0.co;2-4 10619420 PMC6873338

[B10] Folstein M. F. Folstein S. E. McHugh P. R. (1975). “Mini-mental state”: A practical method for grading the cognitive state of patients for the clinician. *J. Psychiatric Res.* 12 189–198. 10.1016/0022-3956(75)90026-6 1202204

[B11] Friederici A. D. (2011). The brain basis of language processing: From structure to function. *Physiol. Rev.* 91 1357–1392. 10.1152/physrev.00006.2011 22013214

[B12] Friedman N. P. Robbins T. W. (2022). The role of prefrontal cortex in cognitive control and executive function. *Neuropsychopharmacology* 47 72–89. 10.1038/s41386-021-01132-0 34408280 PMC8617292

[B13] Fuster J. M. (2000). Executive frontal functions. *Exp. Brain Res.* 133 66–70. 10.1007/s002210000401 10933211

[B14] Goto M. Abe O. Hagiwara A. Fujita S. Kamagata K. Hori M. (2022). Advantages of using both voxel- and surface-based morphometry in cortical morphology analysis: A review of various applications. *Magn. Reson. Med. Sci.* 21 41–57. 10.2463/mrms.rev.2021-0096 35185061 PMC9199978

[B15] Henry J. D. Crawford J. R. Phillips L. H. (2004). Verbal fluency performance in dementia of the Alzheimer’s type: A meta-analysis. *Neuropsychologia* 42 1212–1222. 10.1016/j.neuropsychologia.2004.02.001 15178173

[B16] Im K. Lee J.-M. Won Seo S. Hyung Kim S. Kim S. I. Na D. L. (2008). Sulcal morphology changes and their relationship with cortical thickness and gyral white matter volume in mild cognitive impairment and Alzheimer’s disease. *NeuroImage* 43 103–113. 10.1016/j.neuroimage.2008.07.016 18691657

[B17] Ismail Z. Rajji T. K. Shulman K. I. (2010). Brief cognitive screening instruments: An update. *Int. J. Geriatr. Psychiatry* 25 111–120. 10.1002/gps.2306 19582756

[B18] Jack C. R. Andrews J. S. Beach T. G. Buracchio T. Dunn B. Graf A. (2024). Revised criteria for diagnosis and staging of Alzheimer’s disease: Alzheimer’s association workgroup. *Alzheimer’s Dement.* 20 5143–5169. 10.1002/alz.13859 38934362 PMC11350039

[B19] Jack C. R. Knopman D. S. Jagust W. J. Shaw L. M. Aisen P. S. Weiner M. W. (2010). Hypothetical model of dynamic biomarkers of the Alzheimer’s pathological cascade. *Lancet Neurol.* 9 119–128. 10.1016/S1474-4422(09)70299-6 20083042 PMC2819840

[B20] Javed E. Suárez-Méndez I. Susi G. Román J. V. Palva J. M. Maestú F. (2025). A shift toward supercritical brain dynamics predicts Alzheimer’s disease progression. *J. Neurosci.* 45:e0688242024. 10.1523/jneurosci.0688-24.2024 40011070 PMC11867000

[B21] Jonathan D. Cohen, Alexander L. Nelson, Steven M. Wig (2011). Functional Network Organization of the Human Brain. *Neuron* 72 665–678. 10.1016/j.neuron.2011.09.006 22099467 PMC3222858

[B22] Kang S. H. Park Y. H. Lee D. Kim J. P. Chin J. Ahn Y. (2019). The cortical neuroanatomy related to specific neuropsychological deficits in Alzheimer’s continuum. *Dement. Neurocogn. Disord.* 18 77–95. 10.12779/dnd.2019.18.3.77 31681443 PMC6819670

[B23] Keith C. M. Haut M. W. Wilhelmsen K. Mehta R. I. Miller M. Navia R. O. (2023). Frontal and temporal lobe correlates of verbal learning and memory in aMCI and suspected Alzheimer’s disease dementia. *Neuropsychol. Dev. Cogn. B Aging Neuropsychol. Cogn.* 30 923–939. 10.1080/13825585.2022.2144618 36367308

[B24] Kim H. Na D. L. (1999). Normative data on the Korean version of the Boston Naming Test. *J. Clin. Exp. Neuropsychol.* 21 127–133. 10.1076/jcen.21.1.127.942 10421007

[B25] Kravitz D. J. Saleem K. S. Baker C. I. Mishkin M. (2011). A new neural framework for visuospatial processing. *Nat. Rev. Neurosci.* 12 217–230. 10.1038/nrn3008 21415848 PMC3388718

[B26] Lebed E. Jacova C. Wang L. Beg M. F. (2013). Novel surface-smoothing based local gyrification index. *IEEE Trans. Med. Imaging* 32 660–669. 10.1109/TMI.2012.2230640 23212343

[B27] Liu T. Lipnicki D. M. Zhu W. Tao D. Zhang C. Cui Y. (2012). Cortical gyrification and sulcal spans in early stage Alzheimer’s disease. *PLoS One* 7:e31083. 10.1371/journal.pone.0031083 22363554 PMC3283590

[B28] Lucibello S. Bertè G. Verdolotti T. Lucignani M. Napolitano A. D’Abronzo R. (2022). Cortical thickness and clinical findings in prescholar children with autism spectrum disorder. *Front. Neurosci.* 15:776860. 10.3389/fnins.2021.776860 35197818 PMC8858962

[B29] Luders E. Thompson P. M. Narr K. L. Toga A. W. Jancke L. Gaser C. (2006). A curvature-based approach to estimate local gyrification on the cortical surface. *NeuroImage* 29 1224–1230. 10.1016/j.neuroimage.2005.08.049 16223589

[B30] Lyu I. Kang H. Woodward N. D. Landman B. A. (2018a). Sulcal depth-based cortical shape analysis in normal healthy control and schizophrenia groups. *Proc. SPIE Int. Soc. Opt. Eng.* 10574:1057402. 10.1117/12.2293275 29887663 PMC5992897

[B31] Lyu I. Kang H. Woodward N. D. Styner M. A. Landman B. A. (2019). Hierarchical spherical deformation for cortical surface registration. *Med. Image Anal.* 57 72–88. 10.1016/j.media.2019.06.013 31280090 PMC6733638

[B32] Lyu I. Kim S. H. Girault J. B. Gilmore J. H. Styner M. A. (2018b). A cortical shape-adaptive approach to local gyrification index. *Med. Image Analys.* 48 244–258. 10.1016/j.media.2018.06.009 PMC6167255 29990689

[B33] Milne A. Culverwell A. Guss R. Tuppen J. Whelton R. (2008). Screening for dementia in primary care: A review of the use, efficacy and quality of measures. *Int. Psychogeriatrics* 20 911–926. 10.1017/S1041610208007394 18533066

[B34] Möller C. Vrenken H. Jiskoot L. Versteeg A. Barkhof F. Scheltens P. (2013). Different patterns of gray matter atrophy in early- and late-onset Alzheimer’s disease. *Neurobiol. Aging* 34 2014–2022. 10.1016/j.neurobiolaging.2013.02.013 23561509

[B35] Nichols E. Steinmetz J. D. Vollset S. E. Fukutaki K. Chalek J. Abd-Allah F. (2022). Estimation of the global prevalence of dementia in 2019 and forecasted prevalence in 2050: An analysis for the Global burden of disease study 2019. *Lancet Public Health* 7 e105–e125. 10.1016/S2468-2667(21)00249-8 34998485 PMC8810394

[B36] Płonka O. Krześniak A. Adamczyk P. (2020). Analysis of local gyrification index using a novel shape-adaptive kernel and the standard FreeSurfer spherical kernel — evidence from chronic schizophrenia outpatients. *Heliyon* 6:e04172. 10.1016/j.heliyon.2020.e04172 32551394 PMC7287247

[B37] Power J. D. Cohen A. L. Nelson S. M. Wig G. S. Barnes K. A. Church J. A. (2011). Functional network organization of the human brain. *Neuron* 72 665–678. 10.1016/j.neuron.2011.09.006 22099467 PMC3222858

[B38] Ryu H. J. Yang D. W. (2023). The seoul neuropsychological screening battery (SNSB) for comprehensive neuropsychological assessment. *Dement Neurocogn Disord* 22 1–15. 10.12779/dnd.2023.22.1.1 36814700 PMC9939572

[B39] Sattler C. Toro P. Schönknecht P. Schröder J. (2012). Cognitive activity, education and socioeconomic status as preventive factors for mild cognitive impairment and Alzheimer’s disease. *Psychiatry Res.* 196 90–95. 10.1016/j.psychres.2011.11.012 22390831

[B40] Schaer M. Cuadra M. B. Tamarit L. Lazeyras F. Eliez S. Thiran J. P. (2008). A surface-based approach to quantify local cortical gyrification. *IEEE Trans. Med. Imaging* 27 161–170. 10.1109/TMI.2007.903576 18334438

[B41] Stoebner Z. A. Hett K. Lyu I. Johnson H. Paulsen J. S. Long J. D. (2023). Comprehensive shape analysis of the cortex in Huntington’s disease. *Hum. Brain Mapp.* 44 1417–1431. 10.1002/hbm.26125 36409662 PMC9921229

[B42] Verma M. Howard R. J. (2012). Semantic memory and language dysfunction in early Alzheimer’s disease: A review. *Int. J. Geriatr. Psychiatry* 27 1209–1217. 10.1002/gps.3766 22298328

[B43] Vos T. Lim S. S. Abbafati C. Abbas K. M. Abbasi M. Abbasifard M. (2020). Global burden of 369 diseases and injuries in 204 countries and territories, 1990–2019: A systematic analysis for the Global burden of disease study 2019. *Lancet* 396 1204–1222. 10.1016/S0140-6736(20)30925-9 33069326 PMC7567026

[B44] Weiner K. S. Zilles K. (2016). The anatomical and functional specialization of the fusiform gyrus. *Neuropsychologia* 83 48–62. 10.1016/j.neuropsychologia.2015.06.033 26119921 PMC4714959

[B45] Wig G. S. Laumann T. O. Petersen S. E. (2014). An approach for parcellating human cortical areas using resting-state correlations. *NeuroImage* 93 276–291. 10.1016/j.neuroimage.2013.07.035 23876247 PMC3912214

[B46] Worsley K. J. Taylor J. E. Carbonell F. Chung M. K. Duerden E. Bernhardt B. (2009). SurfStat: A Matlab toolbox for the statistical analysis of univariate and multivariate surface and volumetric data using linear mixed effects models and random field theory. *NeuroImage* 47:S102. 10.1016/S1053-8119(09)70882-1

[B47] Yao J. K. Voorhies W. I. Miller J. A. Bunge S. A. Weiner K. S. (2023). Sulcal depth in prefrontal cortex: A novel predictor of working memory performance. *Cereb. Cortex* 33 1799–1813. 10.1093/cercor/bhac173 35589102 PMC9977365

[B48] Zoltowski A. R. Lyu I. Failla M. Mash L. E. Dunham K. Feldman J. I. (2021). Cortical morphology in autism: Findings from a cortical shape-adaptive approach to local gyrification indexing. *Cereb. Cortex* 31 5188–5205. 10.1093/cercor/bhab151 34195789 PMC8491691

