# Supplementary material for: Local gyrification index and sulcal depth as imaging markers of cognitive decline in Alzheimer’s disease
Source: Front Aging Neurosci. 2025 Aug 12;17:1635861. doi: 10.3389/fnagi.2025.1635861 (PMC12378321; doi:10.3389/fnagi.2025.1635861)
Supplement: Supplementary file 1 [file Table_1.docx]

Supplementary Material

# Supplementary Data

MRI Data Acquisition Parameters

Philips Ingenia CX: 3D T1w images with 0.98 × 0.98 × 1 mm³ voxels were acquired using a spoiled gradient echo sequence with flip angle = 8°, repetition time (TR) = 9.905 ms, echo time (TE) = 4.603 ms, matrix = 224 x 224, and field of view (FOV) = 22 cm.

# Supplementary Tables

## Supplementary Tables

**Supplementary Table 1.** Significant cortical clusters associated with attention function scores.

| Cortical feature | Cluster | Cluster Size (vertices) | Peak t-value | Corrected p-value (FWE) |
| --- | --- | --- | --- | --- |
| SD | Left postcentral and supramarginal gyri | 1606 | 3.39 | 0.025 |
| LGI | Right inferior frontal gyrus | 2149 | 4.70 | <0.001 |
| LGI | Right entorhinal and parahippocampal gyri | 585 | 3.33 | <0.001 |

CT: cortical thickness, FWE: family-wise error, LGI: local gyrification index, SD: sulcal depth

**Supplementary Table 2.** Significant cortical clusters associated with memory function scores.

| Cortical feature | Cluster | Cluster Size (vertices) | Peak t-value | Corrected p-value (FWE) |
| --- | --- | --- | --- | --- |
| CT (positive correlation) | Right parahippocampal gyrus | 520 | 4.57 | <0.001 |
| CT (negative correlation) | Left lateral occipital gyrus | 375 | 4.13 | 0.024 |
| SD (negative correlation) | Left superior frontal gyrus | 455 | 3.89 | 0.023 |
| LGI (positive correlation) | Left insula | 1092 | 3.90 | 0.008 |
| LGI (positive correlation) | Right precentral gyrus | 286 | 3.77 | 0.049 |

CT: cortical thickness, FWE: family-wise error, LGI: local gyrification index, SD: sulcal depth

**Supplementary Table 3.** Significant cortical clusters associated with visuospatial function scores.

| Cortical feature | Cluster | Cluster Size (vertices) | Peak t-value | Corrected p-value (FWE) |
| --- | --- | --- | --- | --- |
| CT | Right inferior and superior parietal gyri | 2835 | 4.83 | <0.001 |
| CT | Left fusiform, lingual, and parahippocampal gyri | 1624 | 6.14 | <0.001 |
| CT | Right cingulate gyrus and precuneus | 1360 | 4.70 | <0.001 |
| CT | Right inferior parietal, middle temporal, and superior temporal gyri | 1274 | 4.22 | <0.001 |
| CT | Right cingulate, paracentral gyri, and precuneus | 1214 | 3.96 | <0.001 |
| CT | Left superior parietal gyrus | 937 | 3.71 | <0.001 |
| CT | Left cingulate, lingual gyri, and precuneus | 840 | 5.24 | <0.001 |
| CT | Right inferior parietal and supramarginal gyri | 736 | 4.40 | <0.001 |
| CT | Right middle temporal gyrus | 712 | 3.99 | 0.006 |
| CT | Right fusiform gyrus | 620 | 3.46 | 0.002 |
| CT | Right inferior and superior parietal gyri | 566 | 4.05 | 0.018 |
| CT | Left supramarginal gyrus | 520 | 3.62 | 0.006 |
| CT | Right supramarginal gyrus | 515 | 4.17 | 0.011 |
| CT | Right inferior parietal, superior temporal and supramarginal gyri | 492 | 3.29 | 0.015 |
| CT | Right middle frontal and superior frontal gyri | 455 | 4.16 | <0.001 |
| CT | Left inferior parietal gyrus | 423 | 3.72 | 0.034 |
| CT | Left cingulate gyrus and precuneus | 418 | 4.12 | 0.046 |
| CT | Right inferior and middle temporal gyri | 417 | 3.28 | 0.006 |
| CT | Left pars opercularis and pars triangularis | 394 | 3.45 | 0.004 |
| CT | Left inferior and middle temporal gyri | 335 | 4.59 | 0.020 |
| LGI (positive correlation) | Left supramarginal and postcentral gyri | 769 | 3.37 | 0.004 |
| LGI (negative correlation) | Right medial and lateral orbitofrontal cortex | 682 | 3.45 | 0.013 |

CT: cortical thickness, FWE: family-wise error, LGI: local gyrification index, SD: sulcal depth
